# Interlayer Coupling Controlled Ordering and Phases in Polar Vortex Superlattices

**DOI:** 10.1021/acs.nanolett.3c03738

**Published:** 2024-02-28

**Authors:** Peter Meisenheimer, Arundhati Ghosal, Eric Hoglund, Zhiyang Wang, Piush Behera, Fernando Gómez-Ortiz, Pravin Kavle, Evguenia Karapetrova, Pablo García-Fernández, Lane W. Martin, Archana Raja, Long-Qing Chen, Patrick E. Hopkins, Javier Junquera, Ramamoorthy Ramesh

**Affiliations:** † Department of Materials Science and Engineering, University of California, Berkeley, California 94720, United States; ‡ Department of Physics, University of California, Berkeley, California 94720, United States; § Center for Nanophase Materials Sciences, Oak Ridge National Laboratory, Oak Ridge, Tennessee 37830, United States; ^∥^Department of Materials Science and Engineering, ^$^Department of Mechanical and Aerospace Engineering, ^▼^Department of Physics, University of Virginia, Charlottesville, Virginia 22904, United States; ⊥ Department of Materials Science and Engineering, Penn State University, State College, Pennsylvania 16801, United States; # Departamento de Ciencias de la Tierra y Física de la Materia Condensada, Universidad de Cantabria, Avenida de los Castros s/n, 39005 Santander, Spain; ∇ Advanced Photon Source, Argonne National Laboratory, Lemont, Illinois 60439, United States; ¶ Molecular Foundry, Lawrence Berkeley National Laboratory, Berkeley, California 94720, United States; ▲ Materials Sciences Division, Lawrence Berkeley National Laboratory, Berkeley, California 94720, United States; ○ Department of Materials Science and Nanoengineering, Rice University, Houston, Texas 77005, United States; ■ Department of Physics and Astronomy, Rice University, Houston, Texas 77005, United States

**Keywords:** ferroelectrics, superlattice, polar topologies, 3D ordering, phase change

## Abstract

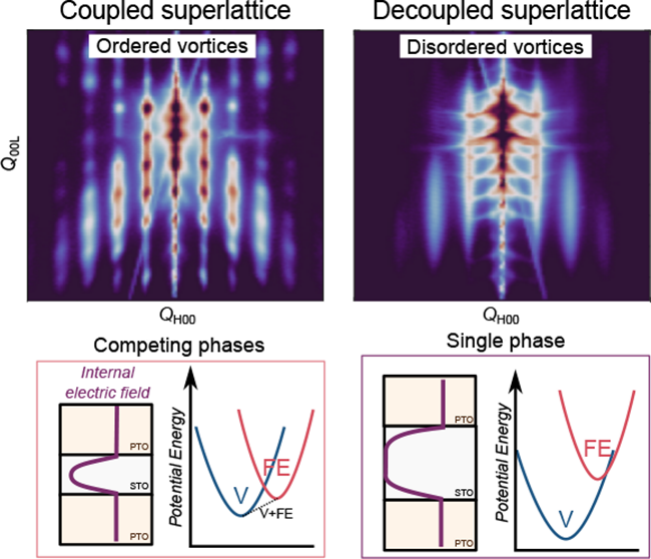

The recent discovery of polar topological structures has opened the door for exciting physics and emergent properties. There is, however, little methodology to engineer stability and ordering in these systems, properties of interest for engineering emergent functionalities. Notably, when the surface area is extended to arbitrary thicknesses, the topological polar texture becomes unstable. Here we show that this instability of the phase is due to electrical coupling between successive layers. We demonstrate that this electrical coupling is indicative of an effective screening length in the dielectric, similar to the conductor–ferroelectric interface. Controlling the electrostatics of the superlattice interfaces, the system can be tuned between a pure topological vortex state and a mixed classical-topological phase. This coupling also enables engineering coherency among the vortices, not only tuning the bulk phase diagram but also enabling the emergence of a 3D lattice of polar textures.

The discovery of nontrivial polarization patterns in oxide thin films has opened a plethora of research avenues, with new phases possessing exotic functional properties such as negative capacitance and controllable chirality.^1,2^ Progress over the past few years has been primarily focused on the model system of thin film ferroelectric–dielectric [(PbTiO_3_)_*n*_/(SrTiO_3_)_*m*_]_*N*_ superlattices (SLs), where *n* and *m* are the number of unit cells and *N* the number of repetitions (Supp. Figure 1). In these structures, the ferroelectric PbTiO_3_ (PTO) layers are subject to electrostatic and mechanical boundary conditions imposed by the dielectric SrTiO_3_ (STO) and the substrate, which stabilize complex polar textures. Since the initial observations of the dipolar structures in [PTO_*n*_/STO_*m*_]_*N*_ superlattices, it has been demonstrated that the ground state strongly depends on (i) the electrostatic boundary conditions, including external electric fields, and (ii) the mechanical boundary conditions, epitaxial or otherwise,^3,4^ which compete in a balanced way leading to phase competition and coexistence.^5,6^

In experimental observations of polar vortices in multilayer (*N* > 1) PTO/STO superlattices, a phase coexistence between the topological vortex phase and a classical ferroelectric *a*_1_/*a*_2_ phase is always reported.^6,7^ The mechanism for this phase segregation is thus far unknown, but it has been shown to scale with the number of repetitions,^8^*N*, eventually disappearing completely when *N* = 1 in (STO)_20_/(PTO)_20_/(STO)_20_ trilayers.^8,9^ This observation implies that phase competition occurs due to coupling between successive ferroelectric layers, relaxing the discontinuities that are required for vortex formation. Previously demonstrated at the mesoscale, one way to tune the magnitude of the depolarization field in ferroelectric heterostructures is through the thickness of the dielectric spacer between ferroic layers.^10^ Additionally, in heterostructures exhibiting classical ferroelectric domains, if the number of unit cells of STO is small enough with respect to the number of unit cells of PTO, the STO layer is expected to become polarized, akin to a proximity effect in ferromagnet–antiferromagnet bilayers or in ferromagnet–superconductor interfaces.^11,12^ This results in a vanishing electrostatic energy cost from polarization mismatch between the two layers.^12^ In the finite limit, then, we see that there is a nonvanishing electric field distribution at the PTO/STO interface which penetrates a characteristic length into the dielectric, playing a role similar to the screening length in the case of conducting electrodes.^13^

We propose that separation between the classical ferroelectric and topological phases can be understood from the perspective of incomplete screening by the dielectric STO layer, even at a length scale of several nanometers length scale. This would lead to electrostatic coupling between adjacent ferroelectric PTO layers. With the hypothesis that the vortex phase is stabilized by the polar discontinuity (and thus the large electrostatic potential energy) at the ferroelectric–dielectric interface, it would follow that the decay length of the resultant internal electric field in the dielectric layer directly determines the stability of the vortex phase. Once the dielectric is reduced to a thickness where the electric field does not completely decay before encountering the field from the opposite interface, it will result in a less stable vortex phase, and the structure will evolve toward a bulk ferroelectric behavior. We expect this incomplete screening by the dielectric layer to both (i) decrease the stability of the vortex phase, leading to the coexistence between ferroelectric and topological phases, and (ii) increase the *z*-direction coherency of the vortices, as the stray electric fields can interact with neighboring objects. Here we modify the phase landscape of PTO–STO structures through electrostatically driven coupling within the ferroelectric superlattice by tuning the thickness of the dielectric layer across this critical decay length.

Samples were deposited by using RHEED-assisted pulsed laser deposition on single crystalline (110)-oriented DyScO_3_ substrates (Methods). X-ray diffraction (XRD) line scans of [PTO_*n*_/STO_*m*_]_6_ [*n* = 16 (Figure 1), *n* = 20 (Sup. Figure 2), and *m* = 10–30] superlattices show the structural evolution of the ground state. This thickness range of *n* has been shown to be the optimal range for vortex formation,^2,5,6,14^ and multiple periodicities are explored to show generalization of the phenomena. When the number of unit cells of STO ranges between *m* = 10–25, XRD scans reveal two sets of superlattice peaks (Figure 1a). The first (marked FE) corresponds to a phase with in-plane polarized ferroelectric domains, and the second (marked V) corresponds to the vortex phase. This superlattice peak doubling has been observed in previous experiments for *n* = *m* = 16 superlattices^6,7^ and arises from the different out-of-plane (00*L*) effective lattice constants of the ferroelectric *a*_1_/*a*_2_(*c*_*FE*_ = 3.92 Å) and vortex (*c*_*V*_ = 3.94 Å) phases. Here, however, we observe that when the thickness of the dielectric layer is increased up to *m* = 30, the peak splitting disappears, indicating that the mixed-phase is no longer stable. Instead, we observe a phase transition to a single vortex phase present in the PTO layers (Figure 1a).

**Figure 1:**
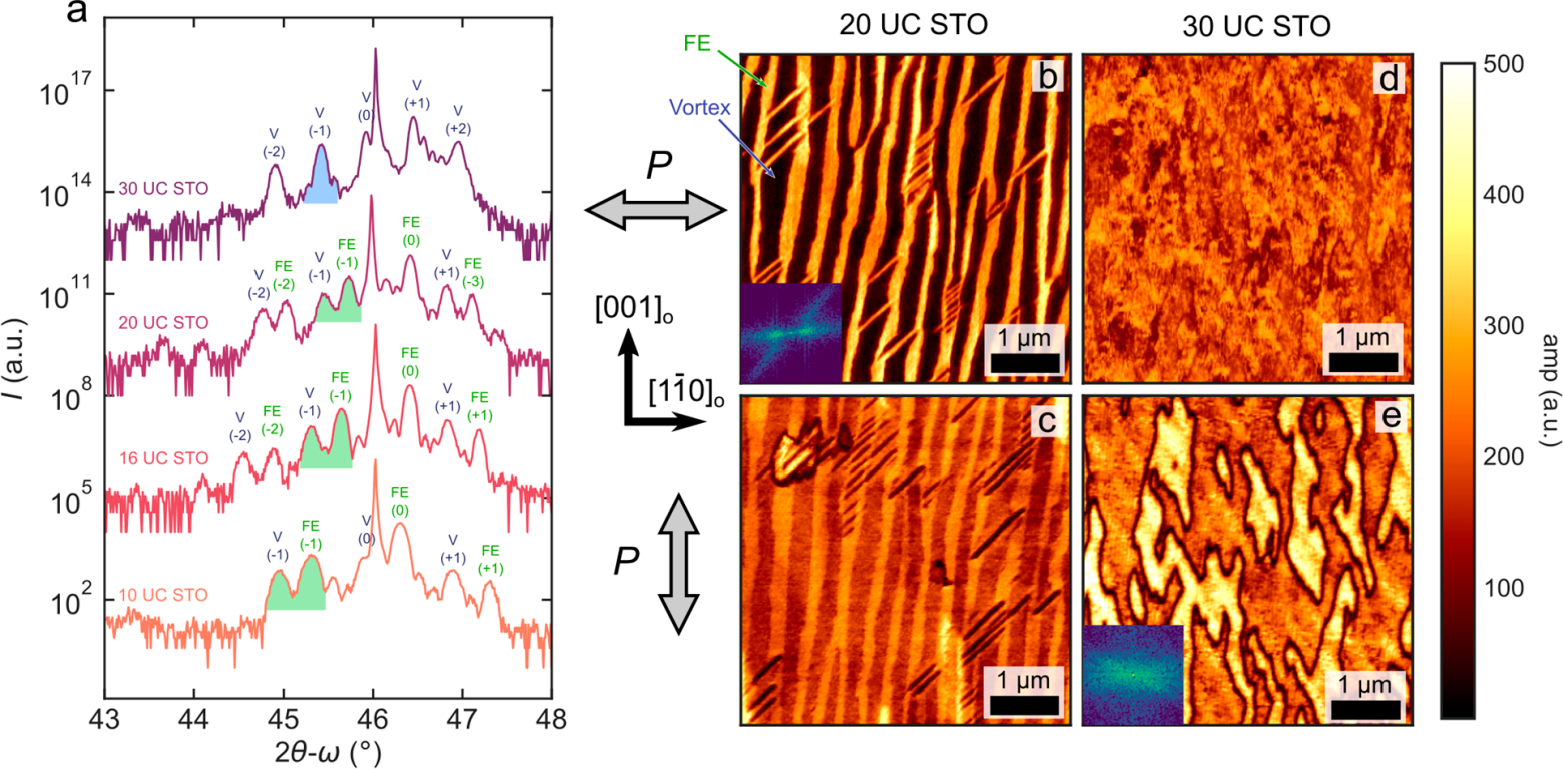
Vortex phase stabilization. (a) High-resolution 2θ–ω scans of [PTO_16_/STO_*m*_]_6_ superlattices grown on DyScO_3_ (110)_o_ for *m* = 10, 16, 20, and 30 unit cells of SrTiO_3_ (bottom to top, respectively). The different peaks associated with the superlattice show the evolution from mixed-phase films, a mixture of ferroelectric *a*_1_/*a*_2_ (FE) with vortex (V), to a pure vortex phase for *m* = 30. The green (blue) shading highlights the double (single) superlattice peak coming from the different (pure) phases. The higher order superlattice reflections are marked with integer numbers. (b–d) PFM amplitudes of superlattices containing *n* = 20 unit cells of PTO and *m* = 20 unit cells (b, c) and *m* = 30 unit cells (d, e) thick STO layers. The vortex axis is along [110]_o_. Top (bottom) panels show in-plane piezoresponse amplitude in the horizontal (vertical) direction, as indicated by the gray arrows at the left side. The vertical stripes in (b) show the phase segregation between the FE a1/a2 (bright) and vortex (dark) phases. These disappear in (d), where the PFM indicates a uniform vortex phase. Insets in (b) and (e) are the corresponding Fourier transforms.

This phase transition can also be observed in in-plane (IP) piezoresponse force microscopy (PFM). When the thickness of the STO is below *m* = 30 unit cells, the characteristic striped ensemble of the mixed *a*_1_/*a*_2_-vortex phase is shown in Figure 1b. Here, the bright contrast corresponds to the stronger IP piezoresponse of the ferroelectric *a*_1_/*a*_2_ phase compared to the small axial polarization component of the vortex phase^9^ along the [110]_o_. In Figure 1c, with polarization measured along the [001]_o_ direction, this contrast between the two phases is partially lost as the *a*_1_/*a*_2_ polarization is now compared to the stronger, lateral polarization component from the buckling of the vortex phase.^9,15^ At a critical thickness *m* ≥ 30 unit cells, this structure condenses into a single vortex phase (Figure 1d) with domains comparable to those previously observed in STO/PTO/STO trilayers.^9^ This is in close agreement with XRD data, and thus we hypothesize that this threshold thickness (*m* = 30) is where the dielectric screening of the STO quenches the interaction between adjacent PTO layers, breaking the bistability of the ferroelectric and vortex phases.

A complementary piece of evidence to demonstrate that this phase mixture is composed of *a*_1_/*a*_2_ and vortex domains can be obtained from second harmonic generation (SHG). In the thin-STO (<30 unit cells) regime, the symmetry of the polar plot agrees well with that expected value from the *P*4*mm* symmetry of the ferroelectric *a*_1_/*a*_2_ phase of PTO, as well as with previous reports on the SHG response of mixed phase PTO/STO superlattices^6,16^ (Figure 2a). In the thick-STO (≥30) regime, the reduced in-plane anisotropy and symmetry of the SHG signal match with what is expected from the pure vortex phase (Figure 2b,c). This indicates that the thin-STO regime is a mixed ferroelectric/vortex structure, with the SHG signal dominated by the higher-intensity ferroelectric phase, while the thick-STO regime is in an approximately pure vortex phase.

**Figure 2:**
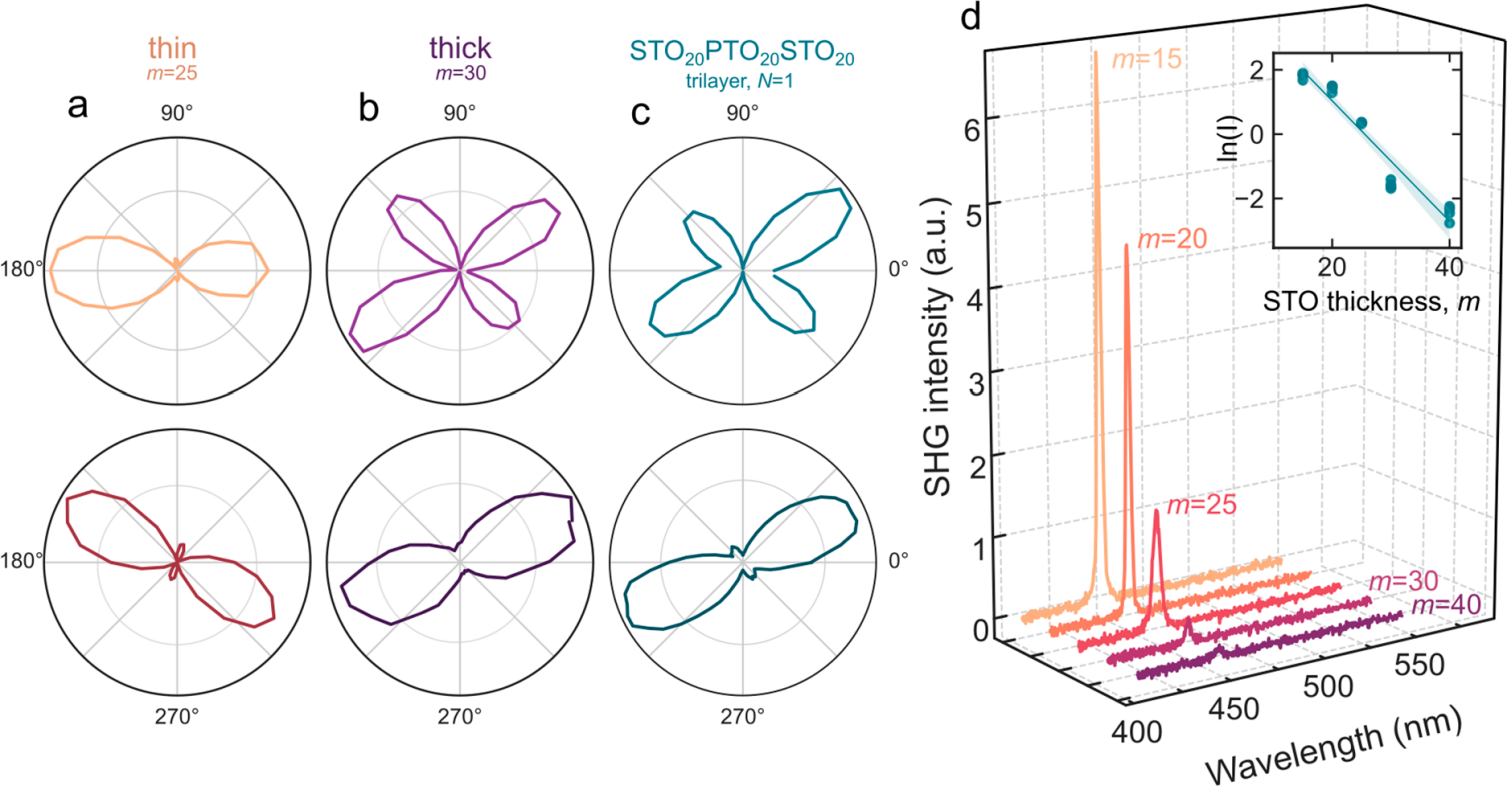
Polarization resolved SHG of the mixed phase. (a, b) Polarization resolved SHG in [PTO_20_/STO_25_]_6_ and [PTO_20_/STO_30_]_6_ superlattices where the analyzer is horizontal (top) and vertical (bottom). These are compared with those of (c), the expected signal from a STO_20_/PTO_20_/STO_20_ trilayer in the pure vortex phase. This shows two distinct regimes that are consistent with previous reports on the mixed ferroelectric/vortex phase (2-fold) when *m* = 25 and the pure vortex phase (pseudo-4-fold) when *m* = 30. (d) SHG spectra as a function of wavelength and STO thickness, *m*, showing the decrease in intensity of the peak at 400 nm as *m* is increased until saturation at *m* ∼ 40 unit cells. Inset shows the natural log of the peak intensity, *I*, against the STO thickness.

SHG in the normal incidence geometry used here is sensitive to broken inversion symmetry in the plane of the film, (110)_o_. Because the ferroelectric *a*_1_/*a*_2_ phase has a stronger in-plane polarization than the vortex phase, SHG intensity should be significantly higher in samples with a higher fraction of the ferroelectric phase.^6^ When the STO layer is thin (*m* < 30), the SHG signal is large (Figure 2d), indicating that the phase fraction of *a*_1_/*a*_2_ domains is large. As the thickness of the STO is increased from *m* = 15 to *m* = 40 unit cells, this intensity decreases continuously until it asymptotes to a small value when the STO is *m* > 30 unit cells thick. SHG intensity is then an approximate phase fraction of the *a*_1_/*a*_2_ phase, scaling exponentially with the thickness of the dielectric. If the phase change is then driven by PTO–PTO coupling in the superlattice, where a thinner dielectric leads to increased electrostatic coupling and a less-stable topological phase, we might expect to see this also reflected in the out-of-plane coherency of the topological structures.

From synchrotron-based reciprocal space maps (RSMs), we observe ∼15 nm period satellites along Q_*0K0*_ that are characteristic of the clockwise–anticlockwise vortex pair periodicity^6,17,18^ (Figure 3a). When the STO thickness *m* < 30 unit cells, there is a clear modulation of the satellite intensity that coincides with the Q_*00L*_ of the superlattice peaks. This Bragg-like enhancement of the satellite intensity is indicative of ordering among the vortices in the out-of-plane (*z*, 00L) direction. As the vortices stack atop one another in the *z*-direction, they take on the same Q_*00L*_ periodicity as the host superlattice and transition from 2D diffraction streaks, which indicate no Q_*00L*_ ordering, to Bragg-like spots when the vortices are completely ordered in *z*. The intensity of these Bragg-like modulations of the satellite peak, Δ_*satellite*_, measured along a line cut at fixed Q_*0K0*_, can then be used to describe the degree of coherence among the vortices. We observe a continuous decrease of the *z*-direction ordering as *m* is increased, tending to Δ_*satellite*_ ≅ 0 when *m* ≥ 30 unit cells thick, when the vortex phase is stable enough to fully outcompete the ferroelectric phase. This continuous evolution from coupled to decoupled can be directly compared to the intensity in the SHG data in Figure 2c, indicating that it is this out-of-plane coupling that is responsible for both ordering of the vortex lattice and the increased fraction of the ferroelectric phase. These critical values of dielectric thickness also match with the phase transition observed in XRD and PFM, where *m* = 30 unit cells marks the transition from the mixed phase to the single vortex phase.

**Figure 3:**
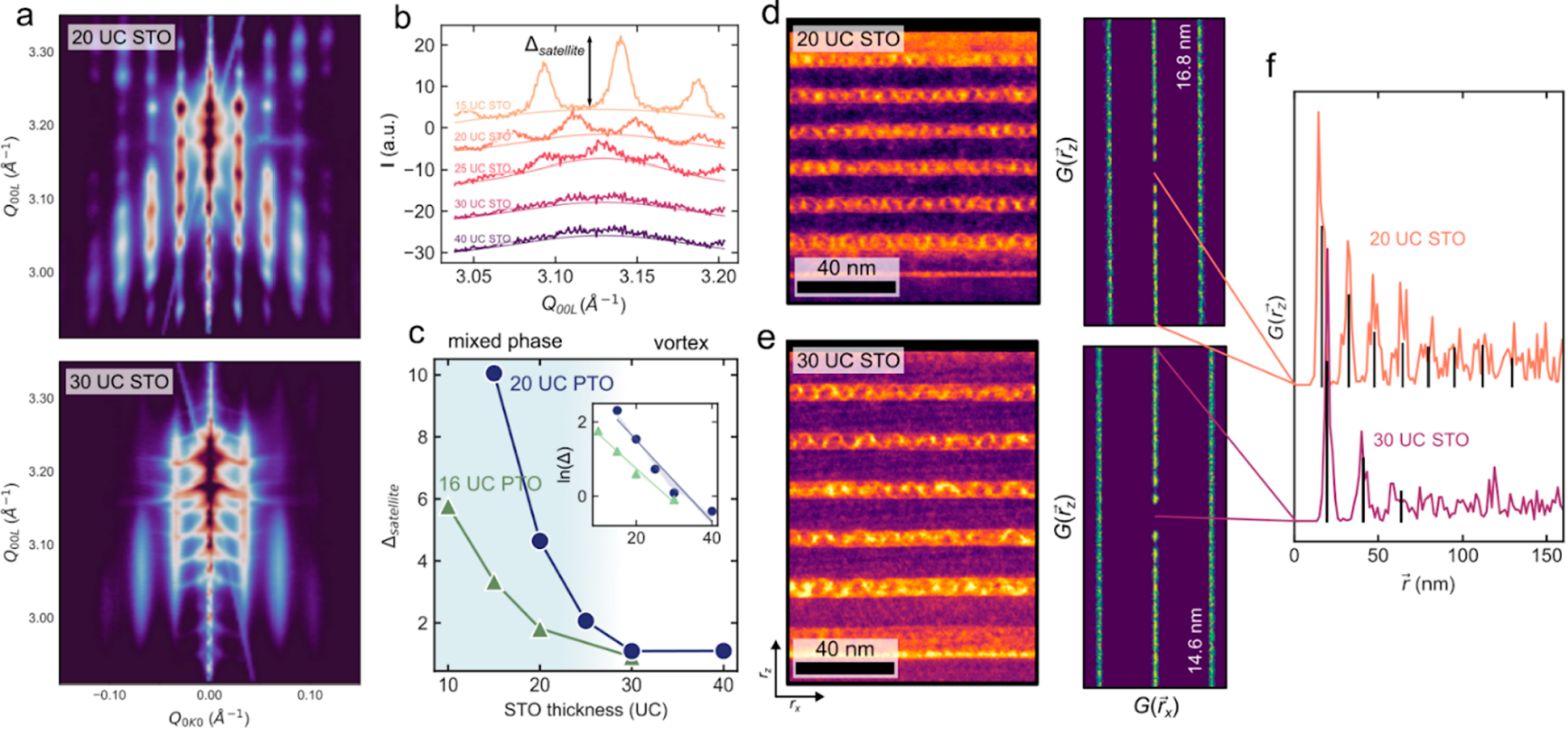
Correlation of the vortices. (a) RSMs of the symmetric 220_o_ (002_pc_) diffraction peak showing Bragg-like modulation of the intensity in the vortex satellites for superlattices with *n* = 20. (b) Line cuts along the *Q*_*00L*_ direction through the first satellite peaks in (a), showing the modulation of intensity, satellite. Fits show the Gaussian background of the satellite itself. (c) Δ_*satellite*_ as a function of STO layer thickness, showing that the *z*-direction coupling between layers decays continuously until ∼30 unit cells. Inset shows ln Δ_*satellite*_ on the same thickness axis. (d, e) LAADF images of the bright PTO layers and dark STO layers in the 20 and 30 unit cell superlattices show diffraction contrast in the PTO layers corresponding to the vortex locations, which are used to calculate the par distribution function, *G*(*r*). The *G*(*r*_*x*_) satellites in the PDFs agree with the *Q*_*0K0*_ spacings observed in the RSMs. (f) *r*_*z*_ line cuts of the PDFs in (d) and (e) showing the slower decay of periodicity in the 20 unit cells STO SL, with 8 oscillations compared to 3, indicating increased ordering in the out-of-plane *z*-direction. Coherent oscillations are highlighted by the black lines.

On a local scale, we can use high- and low-angle annular dark-field scanning transmission electron microscopy (HAADF and LAADF) to directly image the locations of the polar vortices. Quantitatively, by marking the locations of the vortex cores imaged through LAADF, we can reconstruct the pair distribution function (PDF),
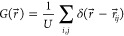
corresponding to the ordering of the vortices (Figure 3d,e). Here, *r⃗* is a vector in real space, *U* is the total number of vortices, and *r⃗*_*ij*_ is the vector between vortices *i* and *j*. *G*(*r⃗*) is the probability of finding a vortex at the corresponding point in space averaged over all vortices. In line cuts along the *r⃗*_*z*_ direction shown in Figure 3f, we can observe the periodicity of the PDF in the *m* = 20 superlattice persists for ∼8 oscillations, and the *m* = 30 superlattice damps after ∼3, indicating that the vortices are more coherent in the *m* = 20 case. This is consistent with quenching of the interaction between neighboring PTO layers occurring only at dielectric thicknesses of *m* ≥ 30 unit cells.

These results help to explain previous observations in polar vortices^8,19,20^ and polar skyrmions,^21,22^ where the topological textures become more coherent with an increasing number of superlattice repetitions. Previously observed in polar skyrmions, for example, there is a scaling of the dielectric response with the number of superlattice units.^22^ These observations point to an origin of either the ferroelectric film thickness, via epitaxial relaxation, or superlattice coupling effects being responsible for the complex domain structure and stability. To rule out epitaxy-dependent thickness effects, a control heterostructure with a thick STO (∼100 nm) buffer layer is deposited on top of an STO_20_PTO_20_STO_20_ trilayer structure on top. In this case (Sup. Figure 2), diffraction satellites and PFM indicate a single-phase vortex structure, the same as grown directly on the substrate. This indicates that the relaxation or presence of epitaxial strain is not responsible for phase segregation, which must be due to the interaction between layers when *m* < 30 unit cells.

The origin of the phase transition from a mixed phase to the pure vortex phase can be traced back to the interplay between electrostatic and elastic boundary conditions.^14,17,18^ Single layer PTO films on DSO substrates, under short-circuit boundary conditions, are stabilized in an *a*/*c* domain configuration.^23,24^ When the structure is then bounded with dielectric STO layers, the *c*-domains are destabilized due to the development of polarization charge at the interfaces, leading to the depolarization field. The electrostatic energy in this configuration can then be minimized in two ways: (i) Rotation of the polarization to the in-plane direction, eventually forming 90° *a*1/*a*2 domain walls. This structure is stabilized by the relatively small energy of such domain walls^25^ and by the tensile strain imposed by DSO over the tetragonal phase of PTO. (ii) The formation of flux-closure domains or continuously rotating polar vortices that minimize the divergence of the polarization at the interface. In PTO/STO superlattices, both mechanisms compete. Here, as the thickness of the STO is reduced, and these interfacial energies change, the vortex phase becomes less stable, and we observe an increasing fraction of the ferroelectric *a*_1_/*a*_2_ phase, validated through phase field and second principles calculations.

In phase field simulations as a function of the dielectric layer thickness, the domain structure observed through PFM can be directly reproduced (Figure 4a,b). When the dielectric layer is below 30 unit cells, the pure vortex phase breaks up into a mixture where stripes of the FE *a*_1_/*a*_2_ phase run perpendicular to the vortex axis (Figure 4a). In these calculations, the free energy can be broken down into its constituent components, and we observe that the phase change is dominated by the exponential increase in the electrostatic energy when decreasing the STO thickness *m* (Figure 4c), which skews the free energy in favor of the *a*_1_/*a*_2_ phase. This phase transition is likely not due to a change in the elastic energy, which follows the opposite trend as the STO thickness is decreased and has a significantly smaller variation across the thicknesses studied here (*E*_*elastic*_ changes by ∼20%, while *E*_*electric*_ changes by more than 100%). This simulated energy follows the same trend and critical values as our experimental measurements of phase fraction and vertical coherency, SHG, and RSMs, supporting the assertion that the decreased screening by the dielectric is responsible for interlayer coupling and phase segregation.

**Figure 4:**
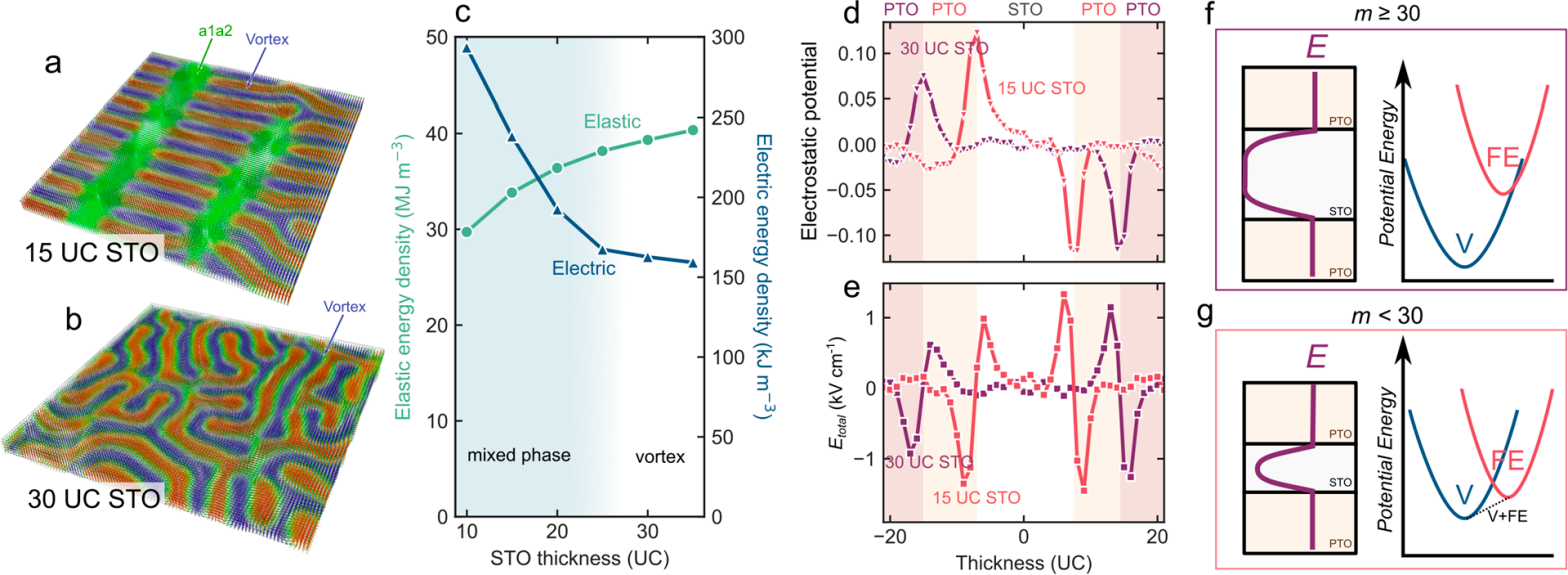
Simulated coupling energies with STO thickness. Phase field simulations of *m* = 15 unit cells (a) and *m* = 30 unit cell (b) superlattices, distinctly showing the coexistence of *a*_1_/*a*_2_ and vortex phases at *m* = 15. (c) Electric and elastic energy densities extracted from phase field simulations. While the electric energy density increases ∼2× as the STO thickness decreases for 30 to 10 unit cells, the elastic energy has the opposite trend and changes by ∼20%, indicating that the electric energy dominates the coupling that leads to the phase transition. (d) Electrical potential and (e) local electric field of *m* = 15 and *m* = 30 unit cell structures determined from second principles as a function of depth, showing the electrostatic discontinuity at the interface and the finite propagation of the electric field into the STO. (f, g) Diagram of the electric field as a function of depth for *m* ≥ 30 and *m* < 30 unit cell structures, showing the proposed mechanism for the electric field coupling between layers. As the layers are coupled and the internal electric field becomes finite, the *a*_1_/*a*_2_ domain structure becomes more stable and competes with the vortex phase, as illustrated by the free energy curves.

Second principles calculations also support our hypothesis that the finite screening length in STO is responsible for the critical thickness. Mapping the electrostatic potential in Figure 4d, we see from second principles that the largest potentials are confined to the first few unit cells of the STO adjacent to the dielectric–ferroelectric interface. From Figure 4e, we can visualize that these interfaces are where the electric field is largest, extending a finite distance into the dielectric layer. When the dielectric layer thickness is then decreased, these interfaces become a larger volume fraction of the layer and dominate the properties of the STO film, decreasing the screening of the electric field proportionally. When this occurs and the electric field within the STO layer becomes finite, the stability of the vortex phase decreases with respect to that of the ferroelectric phase. Eventually, below *m* = 30 unit cells, this results in competition between the two phases and an increasing fraction of the ferroelectric phase. This proposed mechanism is illustrated in Figure 4f,g. This effect could additionally be facilitated by the emergence of a spontaneous polarization in the STO with decreasing thickness, as previous experimental results have described.^26−28^ While there is little experimental evidence that the STO layer in PTO/STO superlattices may be ferroelectric, if the potential barrier between the paraelectric and polar phases is decreased, this could help explain the origin of the enhanced electrostatic coupling.

Here we redefine the conditions for the formation of polar vortices as complete screening by the intermediate dielectric layer. We observe that this screening is a strong function of the thickness of the layer, indicative of a characteristic length similar to the screening length in the case of conducting electrodes.^13^ When screening is incomplete, the classical *a*_1_/*a*_2_ ferroelectric phase becomes competitively stable with the vortex phase, resulting in mixed phase structures. In contrast, however, this coherency of the background electrical potential allows for the out-of-plane ordering of vortex structures between successive superlattice layers, forming a 3D lattice of textures. This generalized guidance helps to explain a number of phenomena observed in polar topological structures and allows for the better design of superlattices with tailored functionalities. By careful engineering of the electrostatics in the superlattice through the properties of the spacer layer, potentially by intentionally introducing a polarization in the ground state, as has been done dynamically with optical excitation,^7^ tuning the degree of ordering in the vortex lattice has important implications for meta-properties driven by their coherent motion.

## Methods

### Thin Film Fabrication

Samples were deposited using pulsed laser deposition monitored with reflection high-energy electron diffraction (RHEED). Samples were deposited at 600 °C in a 100 mTorr O_2_-background pressure using 10 Hz, ∼1.7 J cm^–2^ pulses from a KrF 248 nm excimer laser. Postdeposition, samples were annealed in 30 Torr O_2_ at the growth temperature for 15 min.

Lab-scale X-ray diffraction was performed on a Panalytical diffractometer with a Cu K source. RSMs were taken about the symmetric 220_O_ diffraction peak along the [001]_O_ axis to access the satellite peaks. Synchrotron diffraction was performed at beamline 33BM at the Advanced Photon source at Argonne National Laboratory using a 4-circle goniometer. Samples were imaged using lateral PFM in an asylum MFP-3D at two orthogonal sample rotations to show a ferroelectric domain structure.

### Second Harmonic Generation

SHG measurements were performed using a normal-incidence reflection geometry. A Ti:sapphire oscillator (Chameleon Ultra, Coherent) with ∼100 fs pulses and a center wavelength of 900 nm was used as the excitation source. For control of the polarization a Glan-Thompson polarizer and a subsequent half-wave plate was used. The polarized light was then focused on the sample using a 100× (NA = 0.95) objective. The backscattered signal was sent through a 580 nm short-pass filter and was collected using a spectrometer (SpectraPro 500i, Acton Research Instruments) with a charge-coupled camera (Andor iXon CCD). Emitted light was then sent through a back-end polarizer to select for the polarization of the emitted SHG light. To confirm the incoming and outgoing light polarization, a commercial polarimeter (PAX1000IR1, Thorlabs) was used.

### TEM and Statistics

Cross section STEM samples were made using a Thermo Fisher Scientific Helios Dual Beam focused ion-beam. Initial milling and cleaning were performed at 30 kV, which were sequentially decreased until a finishing energy of 2 kV. STEM samples were imaged on a Thermo Fisher Scientific Themis-Z STEM operating at 200 kV with a 25 mrad convergence angle. LAADF was performed with an inner collection angle of ∼26 mrad, which collects electrons scattered to low angles predominantly resulting in diffraction contrast.

Statistical analysis was performed in python. Vortex locations were found using the scikit-image python package; manual corrections were carried out where necessary. Voronoi tessellation was performed in scipy.

### Phase Field Modeling

See Supp. Note 1.

### Second Principles Modeling

See Supp. Note 2.

## Data Availability

The data that support the findings of this study are available from the corresponding author upon reasonable request.

## Supporting Information Available

Additional data sets for Figures 1–4, including PFM images, XRD spectra, SHG spectra, TEM images, and simulations (PDF)
