# Supplementary material for: Interlayer Coupling Controlled Ordering and Phases in Polar Vortex Superlattices
Source: Nano Lett. 2024 Feb 28;24(10):2972–9. doi: 10.1021/acs.nanolett.3c03738 (PMC10941248; doi:10.1021/acs.nanolett.3c03738)
Supplement: Supplementary file 1 — nl3c03738_si_001.pdf [file nl3c03738_si_001.pdf]

Supporting Information for **Interlayer coupling controlled ordering and phases in polar vortex superlattices**

Peter Meisenheimer*, Arundhati Ghosal, Eric Hoglund, Zhiyang Wang, Piush Behera, Fernando Gómez-Ortiz, Pravin Kavle, Evgenia Karapetrova, Pablo García-Fernández, Lane W. Martin, Archana Raja, Long-Qing Chen, Patrick Hopkins, Javier Junquera, Ramamoorthy Ramesh

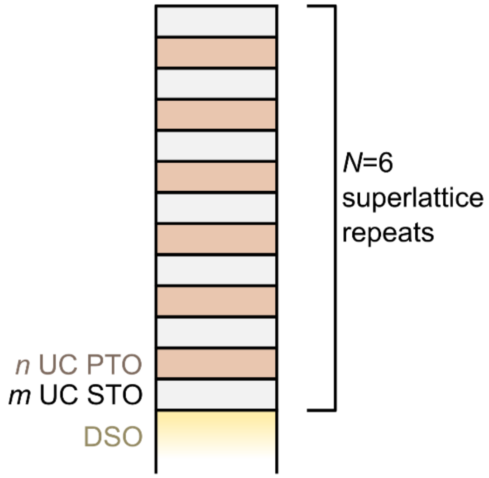

Supp. Figure 1 | Scheme of the $PbTiO_3$-$SrTiO_3$ superlattice studied here, where *n* is the thickness of PTO, *m* is the thickness of STO, and N is the number of superlattice repeats. In the samples studied here, data are shown for both fixed *n* = 16 and 20 UC, which have been previously shown to be the optimal thicknesses for vortices in PTO-STO, where *m* varies from 16 to 40 UC. Thicknesses were determined with RHEED during deposition and confirmed from XRD.

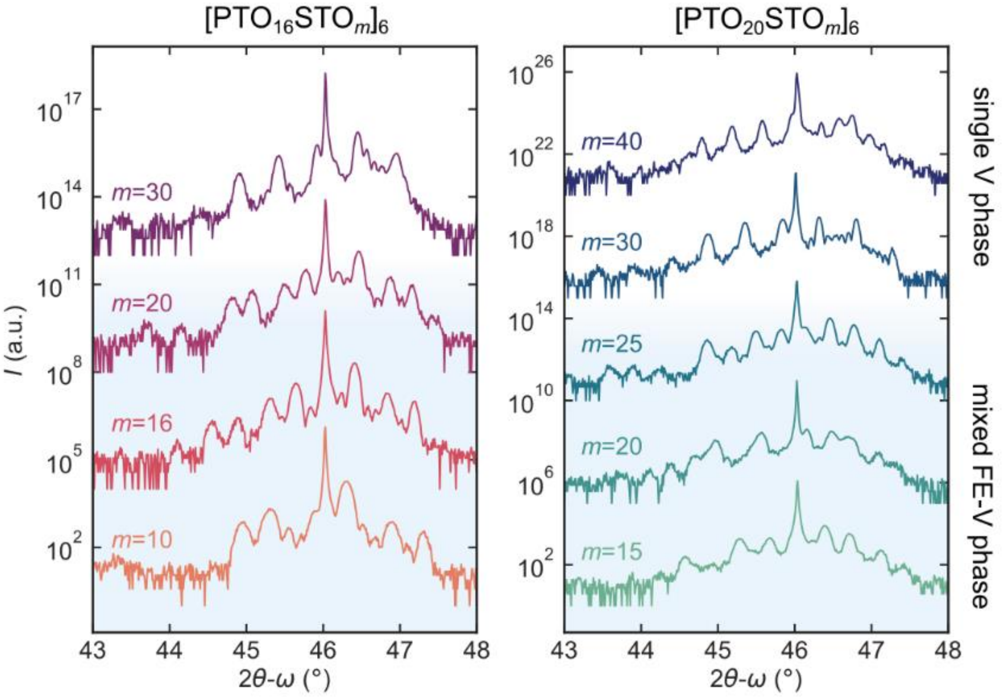

Supp. Figure 2 | XRD 2θ-ω scans of SLs with varying *m* for both *n*=16 and *n*=20 UC thick PTO layers. In both cases the phase mixture disappears at *m*=30. The extra peak at ~46.6 in the *n*=20 *m*=40 is due to a thinner top STO layer to facilitate PFM measurement.

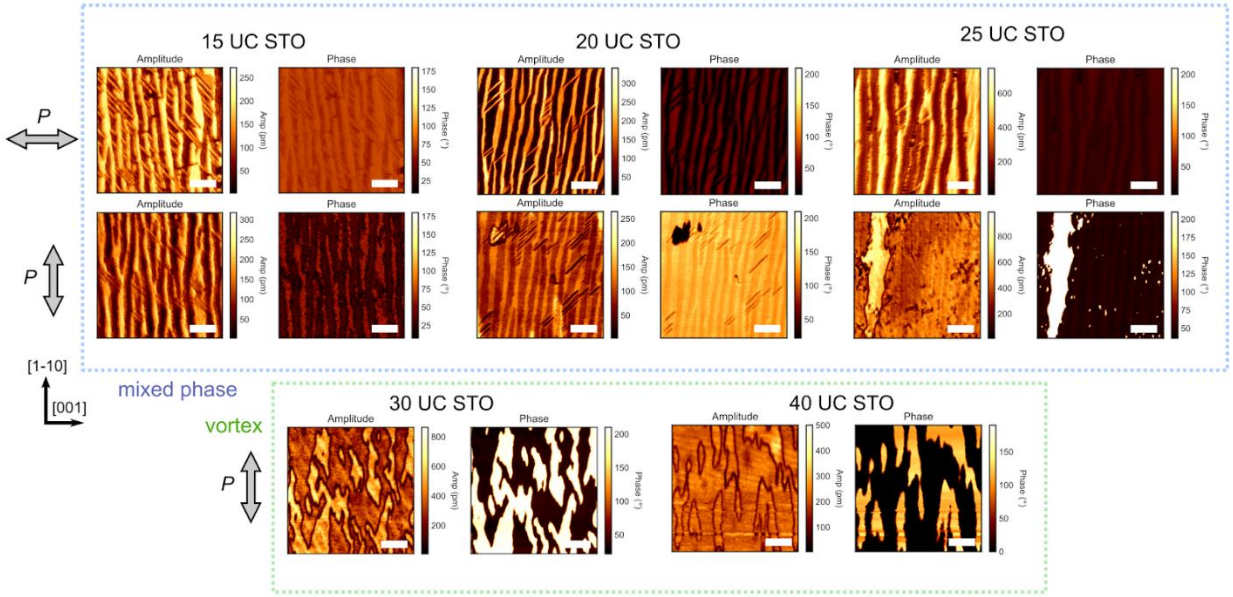

Supp. Figure 3 | Ferroelectric domains in the mixed phase. PFM micrographs showing amplitude and phase along two different in-plane directions shown by the grey arrows. In the mixed phase, when the PTO thickness *n*=20 UC and the STO thickness *m* varies from 15-25 UC, we see the characteristic mixed phase stripes of FE a_1/a_2 that point along $[1\bar{1}0]$. When *m*=30 or 40 UC, we see only the viscous domains characteristic of the buckled variants of the vortex phase. Scale bars are 1 μm.

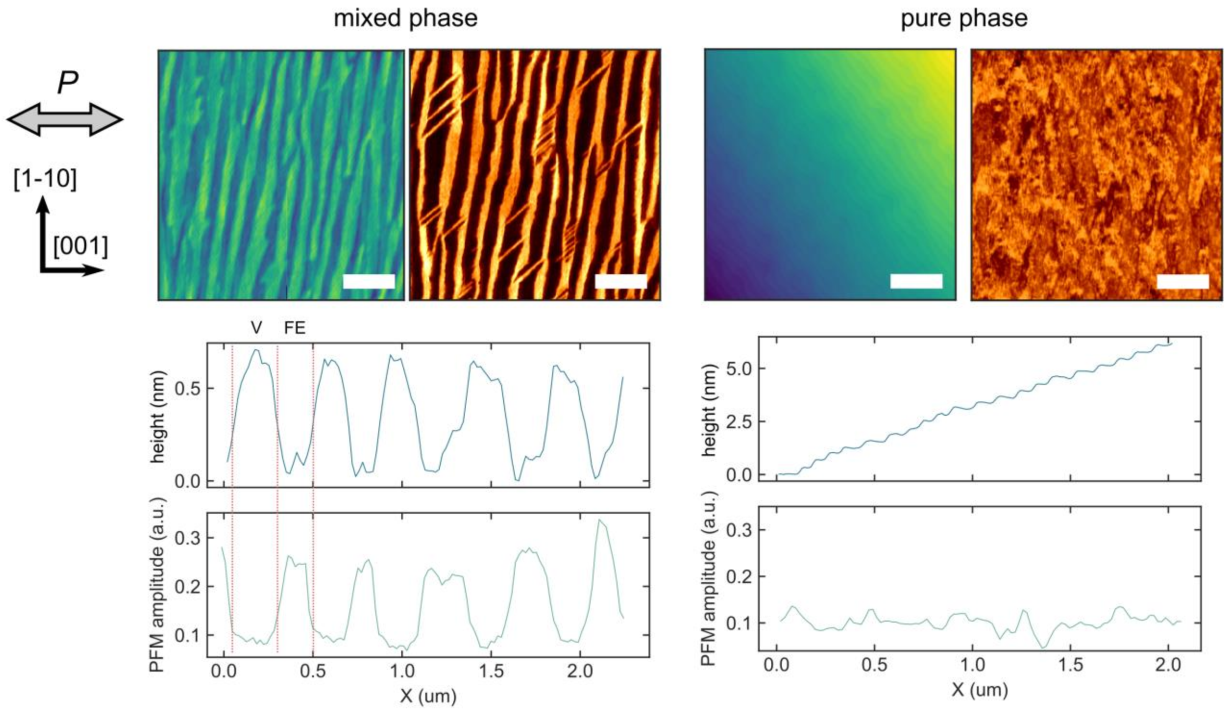

Supp. Figure 4 | Height difference between FE and V. In mixed phase samples, we see a difference in the topography due to the differences in the out-of-plane lattice constants between the two phases (c_{FE} = 3.92 Å, c_V = 3.94 Å). In the taller V phase, the PFM amplitude is lower when measured along the $[001]_o$ direction shown by the gray arrow. In the pure phase sample, the only contrast in topography is due to the faceting of the top STO surface and, in this direction, we see a uniform contrast in the PFM. Scale bars are 1 μm.

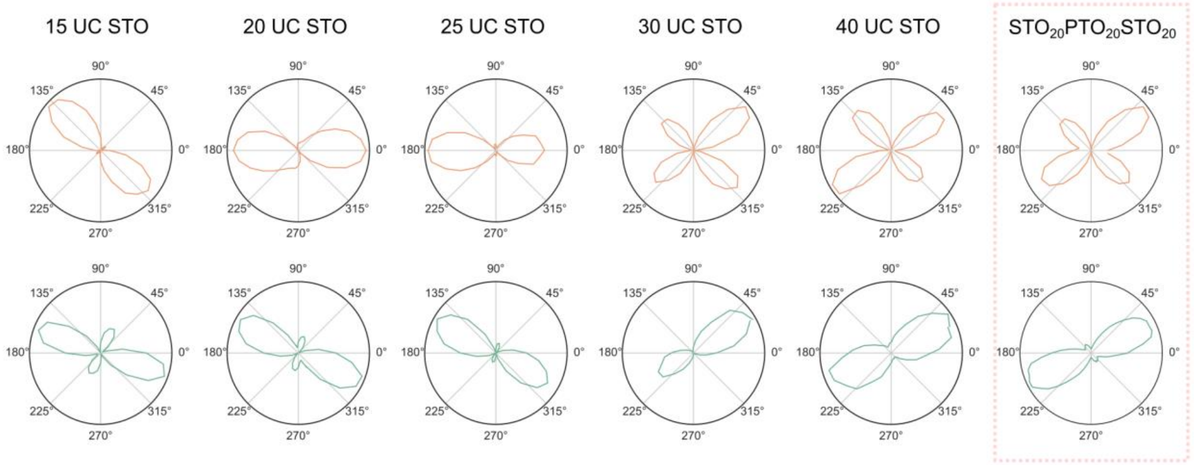

Supp. Figure 5 | Full SHG response. SHG response as a function of polarization when the PTO thickness is fixed to *n*=20 UC and the STO thickness *m* is varied. The final plot is a trilayer, *N*=1, samples, where the symmetry agrees with what is seen in the m>=30 UC cases. The 4-fold symmetry of the plot comes from the two different buckled variants of the vortices [Behera, sciadv]. The sample is excited with 900 nm light and measured at 450 nm. The top row corresponds to when the receiving side polarizer is horizontally oriented, the bottom row vertically.

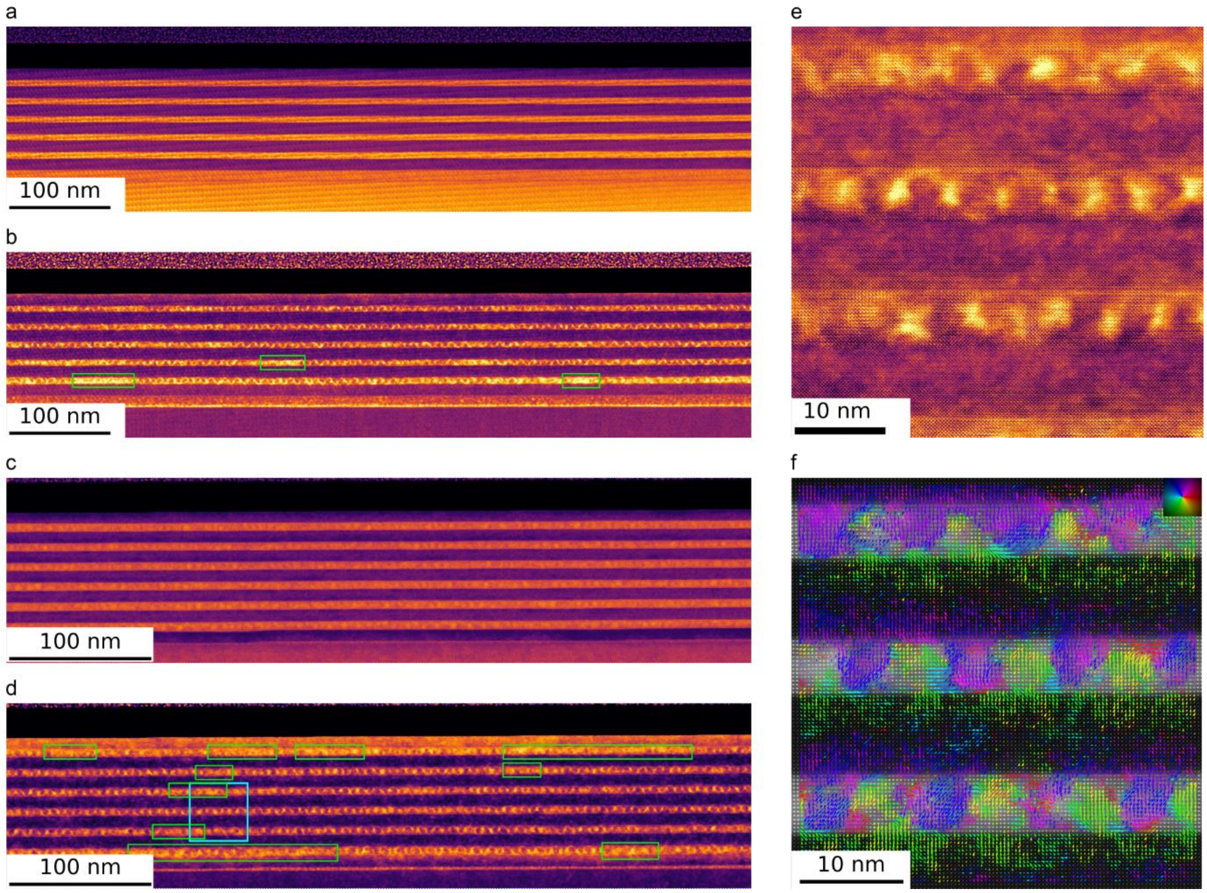

Supp. Figure 6 | STEM HAADF and LAADF of superlattices with *m*=20,30. HAADF images (**a, c**) show the bright PTO layers and dark STO layers in the $[PTO_{16}STO_{30}]_6$, **a**, and $[PTO_{16}STO_{20}]_6$, **c**, SLs. Simultaneously acquired LAADF images (**b, d**) of the *m*=30, **b**, and *m*=20, **d**, SLs show diffraction contrast in the PTO layers. The periodic brighter intensities are from the periodic vortices while regions of continuous bright contrast (marked with green boxes) lack vortices. There is a higher abundance of non-vortex regions in the *m*=20 (**d**). **e** Higher magnification LAADF image from *m*=20 at the boundary of a region containing vortex and non-vortex topologies (cyan marker in d). **f** Displacement map from a drift corrected image in the same region as e, showing larger FE domains in the top left that gradually transitions to vortex ordering as the boundary is traversed to the bottom right.

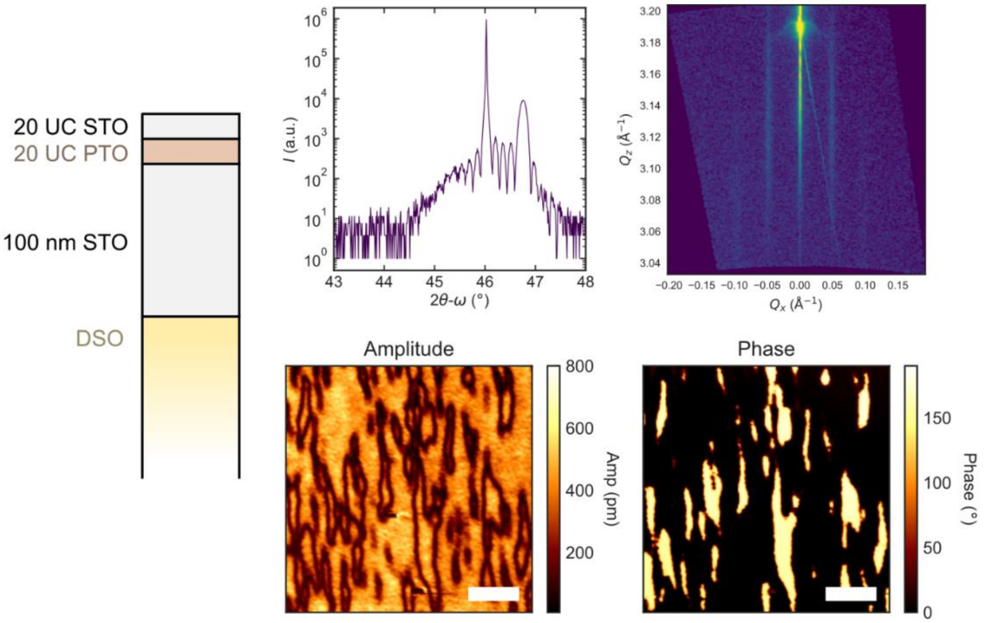

Supp. Figure 7 | Epitaxially relaxed PTO-STO trilayer. $(PbTiO_3)_{20}$-$(SrTiO_3)_{20}$ is deposited on top of 100 nm thick STO, which should relax the majority of the epitaxial strain from the substrate. This sample is in a single phase V state, which indicates that the mixed FE/V phase is due to electrostatic, rather than elastic, forces only when layers are configured in a superlattice.

Supp. Note 1 | Phase field modeling

The phase-field simulation is performed to simulate the polar structure of the (PbTiO3)n/(SrTiO3)m superlattice constrained on the DSO substrate. The polarization $\vec{P}$ is chosen as the order parameter whose time evolution is governed by the time-dependent Ginzburg-Landau equation:

$$\frac{\partial \vec{P}}{\partial t} = -L \frac{\delta F}{\delta \vec{P}}$$

where t is the time, L is the kinetic coefficient. The free energy F is composed of Landau, gradient, elastic, and electric energy:

$$F = \int dV \, f_{Landau} + f_{gradient} + f_{electric} + f_{elastic}$$

Details of the expression and the parameterization of free energy can be found in the previous paper[1,2].

The thickness of (PTO)n is n=10, and the thickness of (STO)m are m=10, 15, 20, 25, 30, 35. The lattice parameters of PTO and STO are set as 3.957 Å and 3.905 Å separately, and the lattice parameters of DSO are set as 3.952 Å and 3.947 Å. A mesh grid of 200Δ*200Δ $* [(n+m) * 3 + m + 40]\Delta$ with $\Delta = 0.4nm$ is used[3]. The thickness dimension consists of 30 layer of substrate, (n+m)*5 layers of the superlattice, and 10 layers of air. In the superlattice layer, m unit cells of $SrTiO_3$ and n unit cells of $PbTiO_3$ are deposited periodically. The periodic boundary condition is employed in the in-plane direction. For out-of-plane direction, the short-circuit electrical boundary condition and the thin film elastic boundary condition is used[4,5]. Random noise with magnitude equals to 0.005 C/m^2 is used as initial condition.

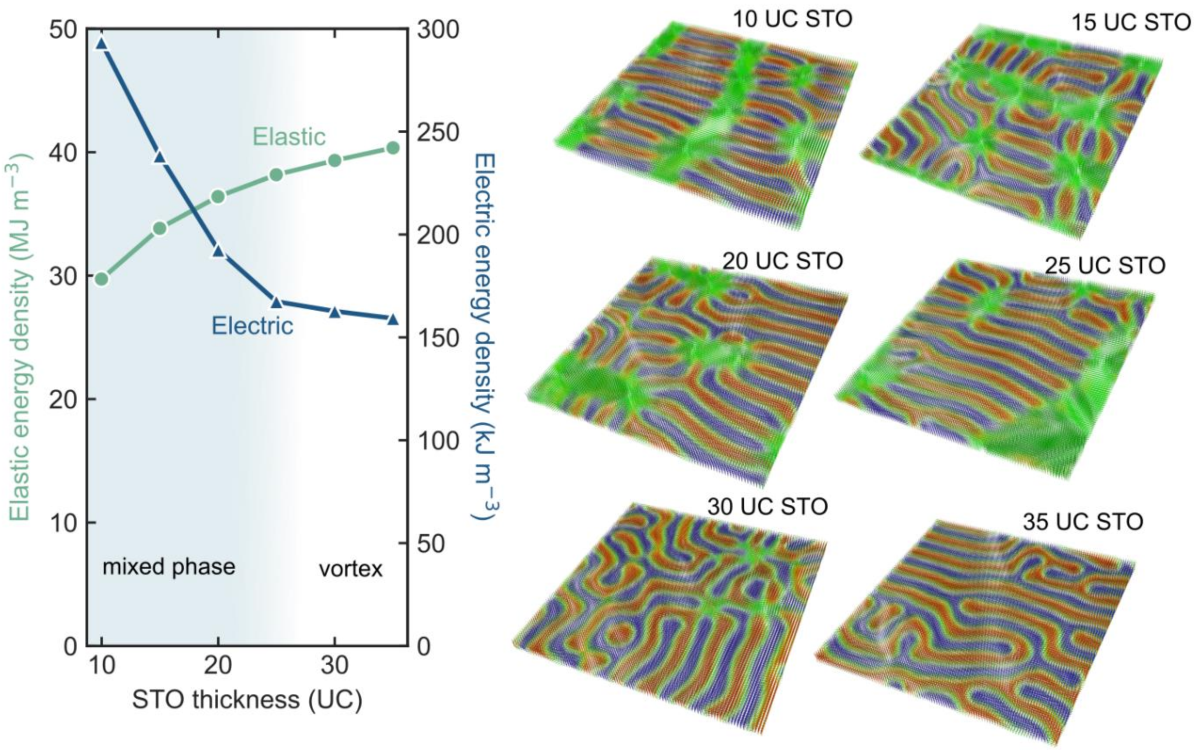

Supp. Figure 8 | Phase field simulations. **a** Electric and elastic energy densities extracted from phase field simulations. While the electric energy density increases ~2x as STO thickness decreases for 30 to 10 UC, the elastic energy has the opposite trend, indicating that the electric energy is dominating the coupling that leads to the phase transition. **b** Example layers of the phase field simulations, illustrating the instability of the V phase when STO is <30UC.

Supp. Note 2 | Second Principles Modeling

The second-principles simulations were performed using the same methodology presented in previous works[6,7] as implemented in the SCALE-UP package[6,8]. The second-principles parameters of both materials were fitted from density functional theory imposing a hydrostatic pressure of -11.2 GPa to counter the underestimation obtained by the local density approximation of the cubic lattice constant that was taken as the reference structure. We imposed an epitaxial constraint assuming in-plane lattice constants of a=b=3.901 Å, forming an angle γ=90º mimicking the conditions of a $SrTiO_3$ substrate. The interatomic potentials, and the approach to simulate the interface, are the ones first introduced in Ref. [7] for a fixed value of $(PbTiO_3)_n$ thickness n=20

several values of $(SrTiO_3)_m$ where studied with m=15, 20, 25, 30. For computational feasibility we have focused on a simulation supercell made of a periodic repetition of 18x2x(n+m). Where L=18 was found to be an stable value to host a two domain structure and two unit cells were considered along the axial direction to account with the antiferrodistortive modes in the $SrTiO_3$ while keeping a computationally affordable superlattice size. We solved the models by running Monte Carlo-simulated annealing from 60 K down to very low temperatures, typically comprising 20,000 relaxation sweeps. Local polarizations are computed within a linear approximation of the product of the Born effective charge tensor times the atomic displacements from the reference structure positions divided by the volume of the unit cell.

References

(1) Li, Y. L.; Hu, S. Y.; Liu, Z. K.; Chen, L. Q. Phase-Field Model of Domain Structures in Ferroelectric Thin Films. *Appl. Phys. Lett.* **2001**, *78* (24), 3878–3880. https://doi.org/10.1063/1.1377855.

(2) Haun, M. J.; Zhuang, Z. Q.; Furman, E.; Jang, S. J.; Cross, L. E. Thermodynamic Theory of the Lead Zirconate-Titanate Solid Solution System, Part III: Curie Constant and Sixth-Order Polarization Interaction Dielectric Stiffness Coefficients. *Ferroelectrics* **1989**, *99* (1), 45–54. https://doi.org/10.1080/00150198908221438.

(3) Hong, Z.; Damodaran, A. R.; Xue, F.; Hsu, S.-L.; Britson, J.; Yadav, A. K.; Nelson, C. T.; Wang, J.-J.; Scott, J. F.; Martin, L. W.; Ramesh, R.; Chen, L.-Q. Stability of Polar Vortex Lattice in Ferroelectric Superlattices. *Nano Lett.* **2017**, *17* (4), 2246–2252. https://doi.org/10.1021/acs.nanolett.6b04875.

(4) Li, Y. L.; Hu, S. Y.; Liu, Z. K.; Chen, L. Q. Effect of Electrical Boundary Conditions on Ferroelectric Domain Structures in Thin Films. *Appl. Phys. Lett.* **2002**, *81* (3), 427–429. https://doi.org/10.1063/1.1492025.

(5) Li, Y. L.; Hu, S. Y.; Liu, Z. K.; Chen, L. Q. Effect of Substrate Constraint on the Stability and Evolution of Ferroelectric Domain Structures in Thin Films. *Acta Materialia* **2002**, *50* (2), 395–411. https://doi.org/10.1016/S1359-6454(01)00360-3.

(6) Wojdeł, J. C.; Hermet, P.; Ljungberg, M. P.; Ghosez, P.; Íñiguez, J. First-Principles Model Potentials for Lattice-Dynamical Studies: General Methodology and Example of Application to Ferroic Perovskite Oxides. *J. Phys.: Condens. Matter* **2013**, *25* (30), 305401. https://doi.org/10.1088/0953-8984/25/30/305401.

(7) Zubko, P.; Wojdeł, J. C.; Hadjimichael, M.; Fernandez-Pena, S.; Sené, A.; Luk'yanchuk, I.; Triscone, J.-M.; Íñiguez, J. Negative Capacitance in Multidomain Ferroelectric Superlattices. *Nature* **2016**, *534* (7608), 524–528. https://doi.org/10.1038/nature17659.

(8) García-Fernández, P.; Wojdeł, J. C.; Íñiguez, J.; Junquera, J. Second-Principles Method for Materials Simulations Including Electron and Lattice Degrees of Freedom. *Phys. Rev. B* **2016**, *93* (19), 195137. https://doi.org/10.1103/PhysRevB.93.195137.
